# Detection of oesophageal cancer biomarkers by plasma proteomic profiling of human cell line xenografts in response to chemotherapy

**DOI:** 10.1038/sj.bjc.6605741

**Published:** 2010-06-15

**Authors:** P Kelly, V Appleyard, K Murray, F Paulin, D Lamont, L Baker, S Suttie, D Exon, A Thompson

**Affiliations:** 1 Centre for Oncology and Molecular Medicine, Ninewells Hospital, University of Dundee, Dundee DD1 9SY, UK; 2 Post-genomics and Molecular Interactions Centre, School of Life Sciences, University of Dundee, Dow Street, Dundee, UK; 3 Department of Surgical Oncology, MD Anderson Cancer Center, 1400 Holcombe Boulevard, Houston, TX 77030, USA

**Keywords:** oesophageal adenocarcinoma, response to chemotherapy, proteomics, biomarkers

## Abstract

**Background:**

The incidence of oesophageal adenocarcinoma is increasing worldwide but survival remains poor. Neoadjuvant chemotherapy may improve survival, but targeting treatment to patients who respond to chemotherapy could be improved by the availability of markers of response. This study sought proteomic markers of therapeutic response using an adenocarcinoma xenograft model.

**Methods:**

Epirubicin, cisplatin or 5-fluorouracil was administered to severe combined immune-deficient mice bearing OE19 oesophageal adenocarcinoma xenografts. Murine plasma samples from treated and untreated xenografts were analysed by surface-enhanced laser desorption/ionisation time-of-flight mass spectroscopy, and panels of peaks were found using class prediction models that distinguished treatment groups. Proteins in these peaks were identified by mass spectroscopy in tryptic digests of purified fractions. Five paired samples from oesophageal cancer patients before and after chemotherapy were analysed using the same methodology.

**Results:**

Plasma protein peaks were identified that differed significantly (*P*<0.05, ANOVA) between the treated xenograft and control groups. Marker panels predicted treated *vs* untreated xenografts with sensitivities of 100%, specificities of 86–100% and test efficiencies of 89–100%. Three of the proteins identified in these panels, apolipoprotein A-I, serum amyloid A and transthyretin were confirmed in the clinical samples.

**Conclusion:**

Plasma protein markers can be detected in response to chemotherapy in oesophageal adenocarcinoma xenografts and in clinical samples, and have the potential to monitor response and guide chemotherapy in oesophageal adenocarcinoma.

The incidence of oesophageal cancer, particularly adenocarcinoma in western populations, is increasing worldwide (Botterweck *et al*, 2000; Park, 2002; Lagergren, 2005) and carries a poor prognosis, even in the minority with resectable disease (Gilbert *et al*, 2002; Munro, 2004) for whom 5-year survival ranges from 10 to 35% (Hulscher *et al*, 2002; Thompson *et al*, 2007). Trials of neoadjuvant chemotherapy and neoadjuvant chemoradiotherapy have reported mixed results ranging from no difference in curative resection or overall survival (Kelsen *et al*, 2007) to improved resection rates and survival (MRC Oesophageal Working Party, 2002; Geh *et al*, 2006). A systematic review of 11 randomised controlled trials showed an increase in overall survival with the use of chemotherapy, but statistical significance was only achieved after 5 years (Maltaner and Fenlon, 2003). Palliative chemotherapy for advanced oesophageal cancer results in control of symptoms in 70–80% of patients with 40–50% objective response rates but only 30–40% surviving for 1 year (Gilbert *et al*, 2002).

The ability to determine, at an early stage, which patients are most likely to respond to chemotherapy could prevent patients undergoing ineffective and potentially toxic treatments and allow direction of treatment to those most likely to benefit. Imaging techniques such as computerised tomography, magnetic resonance imaging, endoscopic ultrasound and positron emission tomography range in their effectiveness to predict response to chemotherapy (Westerterp *et al*, 2005). Pathological criteria for assessment of the degree of tumour regression in the resected oesophagus using tumour regression grades may be a significant predictor of disease-free survival (Mandard *et al*, 1994) but is not an independent prognostic indicator for oesophageal adenocarcinomas (Dunne *et al*, 2001). Pathological response using modified staging criteria has been shown to predict survival following chemoradiotherapy (Swisher *et al*, 2005). In addition, pathological response to pre-operative chemotherapy has been shown to improve overall survival (Kelsen *et al*, 2007). However, neither imaging techniques nor resectional pathology have to date provided robust guidance of response during chemotherapy.

There has been growing interest in the use of proteomic methods on peripheral blood plasma to rapidly profile protein markers that link expression of the genome with the disease process (Cahill, 2001; Plebani, 2005) and to discover novel biomarkers of therapeutic response (Smith *et al*, 2006). Liquid chromatography methods such as high-performance liquid chromatography and two-dimensional liquid chromatography have been increasingly used for protein separation and mass spectroscopy (MS) techniques, such as matrix-assisted laser desorption ionisation time-of-flight, used to analyse the proteins (Colantonio and Chan, 2005). The technique of surface-enhanced laser desorption/ionisation time-of-flight (SELDI–TOF) MS, in which chromatographic separation is achieved on a protein chip surface and analysed intact (Colantonio and Chan, 2005), has been investigated to identify serum and tissue proteomic profiles that could be used in clinical diagnosis.

Surface-enhanced laser desorption/ionisation time-of-flight MS has been reported to differentiate between responders and non-responders treated with chemoradiotherapy (Hayashida *et al*, 2005; Ota *et al*, 2007). In a single study of chemo-radiotherapy in patients with squamous cell carcinoma of the oesophagus, a panel of four peaks was identified, which distinguished chemo-radiotherapy response in 14 out of 15 patients. Although the identity of these markers was not reported (Hayashida *et al*, 2005), the performance of this four-peak panel was superior to radiological and pathological techniques in assessing response to therapy (Ota *et al*, 2007).

In this study, murine plasma protein profiles in mice bearing human oesophageal adenocarcinoma xenografts, treated with three clinically relevant chemotherapy agents: epirubicin, cisplatin or 5-fluorouracil, were used to detect *in vivo* candidate protein markers of response to chemotherapy. The use of a genetically homogenous host eliminates patient variables thus allowing a focus on the effect of the drug on the adenocarcinoma xenografts. The approach taken was to identify markers that differed significantly between normal and xenograft mice and/or differed significantly between treated and untreated xenografts that have potential use as clinical markers of response to chemotherapy. An exploratory study of samples collected from patients with oesophageal cancer before and after chemotherapy was used to test the validity of this approach.

## Materials and methods

### Induction and treatment of xenografts and sample collection

The OE19 oesophageal adenocarcinoma cell line was obtained from the European Collection of Cell Cultures (www.ECACC.org) and cultured in RPMI 1640 cell medium (Pall Laboratories, Portsmouth, UK) under standard conditions. Cells were tested for mycoplasma contamination using the mycoalert test (Cambrex Bio Science, Nottingham, UK), collected for injection, placed in 50% Matrigel (BD Biosciences, Bedford, MA, USA) and administered (100 *μ*l) subcutaneously in the flank of severe combined immune-deficient mice (Harlan, Loughborough, UK). The induction of xenografts was monitored by twice weekly calliper measurements. Groups of mice with established xenografts were injected with clinically equivalent doses of epirubicin (epirubicin hydrochloride; Calbiochem, Darmstadt, Germany) 30 *μ*g per 100 *μ*l (1.5 mg kg^−1^), cisplatin (*cis*-diammine platinum II dichloride; Sigma-Aldrich, Dorset, UK) 48 *μ*g per 100 *μ*l (2.4 mg kg^−1^) and 5-fluorouracil (Fluka, Sigma-Aldrich) 240 *μ*g per 100 *μ*l (12 mg kg^−1^) or control (water, 100 *μ*l) by intra-peritoneal injection once a week for up to three injections (days 0.7 and 14). In addition, groups of control mice not injected with tumour cells received the same doses of drug or water control. At 24 h after the last injection, mice were killed and cardiac blood was collected into heparin plasma tubes for analysis. Samples were separated and plasma was frozen at −70°C before analysis. All xenograft work was performed in accordance with the UK Co-ordinating Committee on Cancer Research guidelines under the requisite home office licences.

### Collection of clinical samples

Samples were collected from five patients with oesophageal cancer (four adenocarcinomas, one squamous cell carcinoma) before and after chemotherapy. Of these five patients, three received neoadjuvant chemotherapy (cisplatin, 5-fluorouracil; two cycles) followed by surgery, one received neoadjuvant chemotherapy (cisplatin, capecitabine; two cycles) followed by chemoradiation therapy and one received palliative chemotherapy (mitomycin C, cisplatin, capecitabine; three cycles). Pathological response following surgery showed a partial response (tumour regression grade 3 or 4) in three of four patients. Samples were separated and plasma was frozen at −70°C before analysis. The patients were enrolled in the study following written informed consent (Grampian Local Research Ethics Committee).

### Analysis of plasma samples by SELDI–TOF MS

Samples from each individual were denatured in 9 M urea and tested at a final dilution of 1 : 100 on CM10 (weak cation exchanger) protein chips pre-equilibrated with 50 mM citrate (pH 4) and Q10 (strong anion exchanger) protein chips pre-equilibrated with 50 mM phosphate (pH 6). The chips were washed and sinapinic acid was added as matrix according to the manufacturer's recommended methods (Bio-Rad, Hemel Hempstead, UK), analysed using a SELDI–TOF MS PSII instrument (Bio-Rad) with an all-in-one protein standard (Ciphergen, Fremont, CA, USA) included on each run. Spectra for each individual were collected over a low molecular weight range (2000–30 000) and high molecular weight range (20 000–150 000) using fixed (optimised) laser intensity and detector sensitivity settings. Data collected from multiple points on each spot were collated into averaged spectra using the instrument software. The spectra from each experiment were normalised using the total ion current and calibrated from the all-in-one protein standard data. Peaks were detected using the instrument's biomarker software, using a peak threshold of 20%, a first pass signal/noise ratio of 5 and a second pass signal/noise ratio of 3. The reproducibility of this method, assessed by repeat testing of a sample yielded typical CVs of 20% in the low molecular weight analyses and 30% in the high molecular weight analysis, was consistent with reported data (Semmes *et al*, 2005). Sample statistics were generated for peaks identified using the biomarker software. For each sample group test performed, a list of peak intensities analysed and *P*-values (ANOVA) obtained was recorded. The above procedure was followed for the analysis of both human and murine samples. An additional analysis was performed on samples from xenografts to correct for the effect of tumour burden. Spectra were selected from untreated and treated xenografts that were matched for tumour volume. Sample statistics were generated to test for significant differences in peak intensities between treated and untreated xenografts (for each drug) in these matched groups. All peaks with *P*<0.05 (95% confidence limit) were considered to be significant. The murine xenograft data were further analysed using pattern recognition software (Genespring GX; Agilent Technologies, Santa Clara, CA, USA). Following published methods (Villanueva *et al*, 2007), we performed an unsupervised hierarchical clustering according to peak *m*/*z* and condition using principal component analysis to detect clustering in relation to each treatment group. To control for non-specific effects of drug treatment that were unrelated to tumour response, we excluded data for peaks that differed significantly between treated and untreated non-xenografts from the analysis and class prediction was performed using *K*-nearest neighbours and Support Vector Machine using peak *m*/*z* lists created by one-way ANOVA, ranked by *P*-value and delimited using *P*-value cut-offs. This ensures that only the most highly significant markers are included in each panel and the cut-off can be varied to obtain the optimal class prediction model with the smallest number of panel members. For each test parameter the class prediction model was optimised using a training set comprising ∼75% of the samples, and validated using an independent test set comprising ∼25% of samples, both sets comprising all treatment groups.

### Protein identification

Peaks from one or more panels that predicted treated *vs* untreated xenografts were selected for protein identification by fractionation followed by MS/MS. Murine samples containing high intensity peaks to be identified were denatured in 9 M urea and fractionated using Q Ceramic Hyper D spin columns equilibrated in 1 M urea (pH 9) and eluted in buffers ranging from pH 9 to pH 3, followed by an organic solvent (columns and buffers all sourced from Bio-Rad). Eluted fractions were spotted on to protein chip surfaces (NP20, H50, Q10 (pH 4) and CM10 (pH 6)) and further analysed by SELDI–TOF MS to determine the fractions containing the target peak (*m*/*z*). Further murine and human samples were fractionated using an off-gel fractionator fitted with 24 cm IPG strips according to the manufacturer's instructions (Agilent Technologies). Selected fractions were concentrated and desalted in 10 mM HEPES using molecular weight cut-off filters (Vivaspin; Sartorius, Goettingen, Germany). The retentates were tested by SELDI MS using CM10, Q10, H50 and/or NP20 protein chips to confirm the optimal location of the peak of interest. The desalted fractions were reduced in 10 mM DTT (10 min incubation at 70°C), alkylated in 50 mM iodoacetamide (30 min incubation at room temperature) and loaded onto nu-PAGE 10–20% Tricine, 10 or 12% Bis-Tris gels (Invitrogen, Carlsbad, CA, USA) with MES running buffer and run with SeeBlue Plus 2 pre-stained standards (Invitrogen). Electrophoresis was performed at 125 V for 70–90 min (Tricine gels) or 200 V for 35–40 min (Bis-Tris gels). Gels were fixed with 50% methanol/10% acetic acid and staining was performed with Coomassie G-250 colloidal stain (Invitrogen) for 3–12 h and destained with deionised water overnight. Selected protein bands were excised from gels using a Harris Unicore 1 mm cutter (Sigma-Aldrich). Proteins were passively eluted from the gel pieces to confirm the presence of the peaks of interest by SELDI MS using CM10, Q10, H50 and/or NP20 protein chips. Matched gel pieces were processed and in-gel digested with Trypsin (Roche, Welwyn, UK) and an aliquot of the digest analysed by nLC-MS-MS using a 4000 QTRAP (Applied Biosystems, Carlsbad, CA, USA). The tandem MS data generated from the observed peptides were analysed and identified using the Mascot search engine (www.matrixscience.com) against the NCBInr and IPI Mouse databases. Only peptides that had ion scores above the significance threshold were reported and grouped into their respective protein identifications, with MOWSE scores indicating probability of correct identification. Peptide sequences obtained were mapped on the known protein sequence to determine the percentage of the sequence covered. Proteins identified with less than two peptides and ion scores below the significance threshold were subsequently confirmed and validated by targeted nLC-MS-MS on an LTQ Orbitrap XL Ion Trap (Thermo Fisher Scientific, Loughborough, UK). In this mode tryptic peptides of known *m*/*z* values are generated from the theoretical protein sequence and then used as a target list during nLC-MS-MS analysis. Duplicate fractionation and analyses were undertaken to confirm and validate the identification of peaks with initial low probability ion scores.

## Results

**Figure 1:**
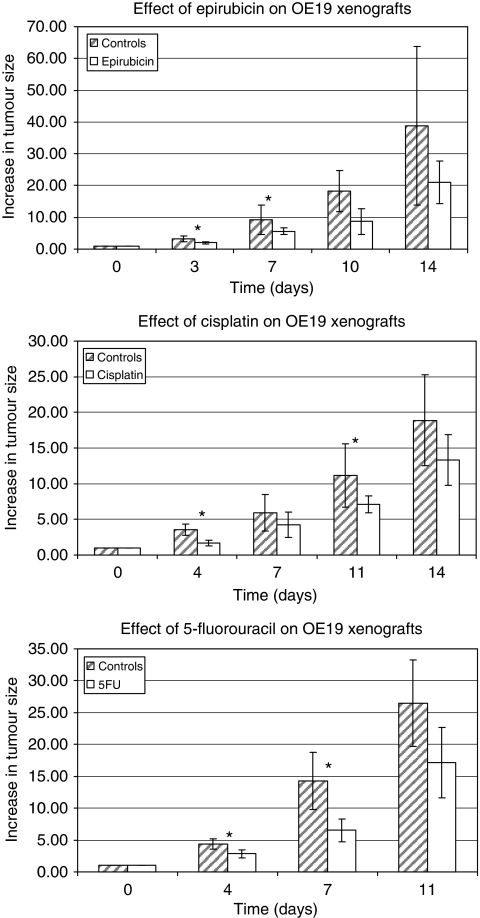
Tumour volume data obtained during treatment of OE19 xenograft bearing mice with each drug (epirubicin, *n*=20, cisplatin, *n*=16 and 5-fluorouracil, *n*=18 or control (blank, *n*=23, 20 and 12 respectively). Mean tumour volumes normalised to day zero (first day of drug injection) and 95% confidence intervals are shown and statistically significant differences (*P*<0.05, two-tailed *t*-test or Mann–Whitney test as appropriate) are highlighted (^*^).

Oesophageal adenocarcinoma OE19 xenografts treated with cisplatin, epirubicin or 5-fluorouracil showed significant growth reduction with all three drugs when compared with matched untreated controls (Figure 1), at the time points indicated (*P*<0.05, two-tailed *t*-test, assuming equal variance or Mann–Whitney test as appropriate).

**Figure 2:**
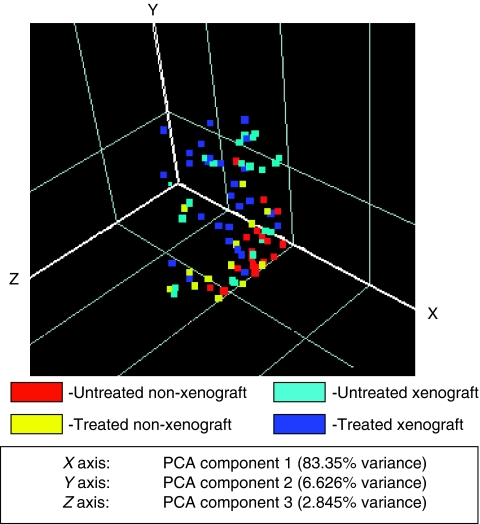
Principal component analysis of plasma profiles obtained in the mouse xenograft experiments. Each point represents a single sample, colour coded as indicated according to untreated xenograft (epirubicin, cisplatin or 5-fluorouracil) treated xenograft, untreated non-xenograft or treated non-xenograft mouse.

Profiles of the murine plasma sample proteins obtained using SELDI–TOF MS on the two (CM10 and Q10) chip surfaces allowed detection of 189 peaks that were present in most samples. A preliminary assessment of this data indicated that statistically significant (*P*<0.05) differences were observed for multiple peak intensities between xenografts and controls, treated and untreated xenografts as well as treated and untreated non-xenografts for all three drug treatments. Significant differences were detected between treated and untreated xenografts that were matched for tumour volume at the time of sample collection, indicating that these differences in response to therapy were not simply caused by changes in tumour burden. Pattern recognition software was therefore used to further elucidate these findings. An initial assessment by principal component analysis performed on this data set (with principal components *X*, *Y* and *Z*) and plotted as a three-dimensional map showed unsupervised clustering of samples according to treatment group (Figure 2). The data were therefore further analysed to determine whether panels of markers could be used to predict the class (treatment group) of each sample. To control for non-specific effects of drug treatment that were unrelated to tumour response, we excluded data for peaks that differed significantly between treated and untreated non-xenografts from this analysis. The most highly significant markers were selected in these panels, by ranking according to *P*-value and application of a *P*-value cut-off. The class prediction results are summarised in Table 1 with the number of (statistically significant, *P*<0.05, following *P*-value cut-off selection) peaks selected for the class prediction model indicated for each test performed. Following optimisation of the *k* nearest neighbours and the Support Vector Machine algorithms using the training set, we tabulated the output obtained for the independent test set using the optimal model in terms of the number of samples correctly predicted and the number of samples incorrectly predicted. From these predictions, calculated sensitivity, specificity, positive and negative predictive value (PPV and NPV) and overall test efficiency for xenograft-bearing mice *vs* mice without xenografts (Table 1a) and treated *vs* untreated xenografts for each drug (Table 1b) show the test method accuracy using the selected panels of protein peaks.

Fractionation of the murine plasma to isolate the protein peaks selected from the class prediction modelling and protein fingerprint analysis of the tryptic digests of these isolated protein peaks resulted in the identification of seven proteins (Table 2), with high MOWSE probability scores and sequence coverage ranging from 24 to 84%. The sequence data indicated that each of these proteins was murine in origin. The proteins identified include markers selected in the class prediction models as annotated (Table 2). Hence this table includes markers that predict presence of tumour and/or response to chemotherapy.

Analysis of the human sample SELDI–TOF MS spectra resulted in the detection of a number of peaks that differed significantly (*P*<0.05) between the pre-chemotherapy and post-chemotherapy samples. Fractionation of the human samples and protein fingerprint analysis of these peaks resulted in the identification of four proteins (Table 3) with high MOWSE probability scores and sequence coverage ranging from 42 to 87%. Three of the eight proteins (serum amyloid A (SAA), transthyretin and apolipoprotein A-1) identified in the murine plasma were confirmed in the human samples, with the identification of one additional protein (retinol-binding protein 4) in the human samples only. Of these four markers, three were increased after chemotherapy and one, SAA, decreased.

## Discussion

The ultimate intent of this research programme is to develop biomarkers predictive of response to therapy in oesophageal adenocarcinoma. The primary objective of this study was to detect candidate circulating biomarkers in mice bearing oesophageal adenocarcinoma xenografts. Proteins were identified that could distinguish groups of xenograft *vs* non-tumour-bearing mice and/or treated and untreated xenograft-bearing mice. As such, the candidate markers have potential as diagnostic markers of oesophageal cancer and/or markers of response to therapy. A pilot study using clinical samples confirmed that three of these candidate markers changed significantly after chemotherapy.

The xenograft model of adenocarcinoma used clinically equivalent doses of epirubicin, cisplatin and 5-fluorouracil, which were effective at reducing growth in the OE19 xenografts (Figure 1). Analysis of the plasma samples taken from the mice by SELDI–TOF MS enabled the identification of multiple peaks that differed significantly between xenograft-bearing and control mice, or between treated and untreated xenograft-bearing mice. Differences between treated and untreated xenografts were evident after these groups were matched for tumour burden at the time of sample collection, indicating that the observed differences reflected response to chemotherapy. Further analysis of the plasma profiles gave evidence of clustering within treatment groups, and class prediction models clearly showed that a panel of peaks was able to discriminate between xenograft-bearing mice and controls, with sensitivity of 92% and specificity of 100%. In addition, unique panels of peaks were able to discriminate between treated and untreated xenografts for each drug (Table 1), with sensitivities of 100% and specificities of 86–100%. Hence, the observed response of human oesophageal adenocarcinoma xenografts to chemotherapy was associated with detectable changes in plasma protein profiles and further work focused on identifying the protein peaks represented in the treatment class prediction models.

The protein peaks identified in the mouse xenograft samples were well-characterised circulating murine proteins. This suggests we have identified host rather than tumour proteins that indicate the presence of tumour and/or response to chemotherapy. None of the proteins reported in this study has previously been related to therapeutic response in oesophageal cancer. These proteins are high rather than low-abundance proteins, as has commonly been the case in proteomic studies of serum or plasma in which the top 20 most abundant proteins represent some 98% of total protein. In this study the decision was taken not to immunodeplete the samples of high-abundance proteins, to ensure that all candidate markers were considered, given the observation that these immunodepletion methods can also deplete samples of low abundance markers that are associated with the abundant carrier proteins (Granger *et al*, 2005).

Apolipoprotein A-1 was in the xenograft *vs* non-tumour-bearing mouse class prediction panel as well as the panels for epirubicin or 5-fluorouracil-treated *vs* -untreated xenografts, suggesting an abnormality in the presence of xenograft that is corrected by treatment. Although this proteomic response is novel in the context of oesophageal cancer, apolipoprotein A-I has previously been reported as a serum marker for the detection of cancer, with a reduction in breast (Huang *et al*, 2006), pancreatic (Ehmann *et al*, 2007) and colorectal (Engwegen *et al*, 2006) cancers when compared with healthy controls. Apolipoprotein C-III was in the panel for 5-fluorouracil-treated *vs* -untreated xenografts. Changes in other apolipoproteins, including apolipoprotein C-III, have also been reported (Huang *et al*, 2006). Like apolipoprotein A-1, transthyretin was in the panel for the xenograft *vs* non-tumour-bearing mice as well as for epirubicin or cisplatin treated *vs* untreated xenografts. Transthyretin (also known as thyroxin-binding prealbumin) has also been reported as a marker, decreased for ovarian (Zhang *et al*, 2004) and increased for lung (Maciel *et al*, 2005) cancer. *β*-2-Microglobulin was in the panel for epirubicin-treated *vs* untreated xenografts. This marker has been proposed as a prognostic marker for myeloma (Jacobson *et al*, 2003).

Serum amyloid A was in the panel for xenografts *vs* non-tumour-bearing mice but not in the panels for treated *vs* untreated xenografts. Increased SAA, an acute-phase protein produced by hepatocytes of the liver (Raynes *et al*, 1991), has also been identified as a significant marker in various cancers including lung (Dowling *et al*, 2007), nasopharyngeal (Liao *et al*, 2008) and gastric (Wang *et al*, 2008) carcinomas by many techniques including MS and immunoassay. Two other markers included in the panel for xenograft *vs* non-xenograft mice were identified as haemoglobin chains. Changes of haemoglobin have been reported previously for breast cancer (Huang *et al*, 2006). Tumour anaemia is a common symptom in cancer patients and haemoglobin levels are associated with survival for ovarian (Obermair *et al*, 1998) and lung (Tomita *et al*, 2008) cancers.

Thus, the proteins identified by the present studies have been suggested as cancer markers for many tumour types, but have not previously been reported as markers for oesophageal cancer. The initial finding that three of the markers identified in the mouse xenograft model were also identified in patient samples confirms the benefit of the experimental approaches used. Although at present SAA, transthyretin and apolipoprotein A-1 as individual markers may be too non-specific to influence therapeutic decisions, taken together, these plasma proteins may represent a panel of circulating markers that provide information about response to therapy in oesophageal cancer. To date we have only looked at a relatively small number of patients with some evidence of pathological response but have not been able to compare the biomarker pattern observed with other indicators of clinical response. Prospective testing of these plasma proteins as markers of response to chemotherapy within clinical trials, comparing responders to non-responders, is now required.

## Figures and Tables

**Table 1:** Summary of class prediction data

**(a) OE19 xenograft-bearing mice *vs* non-xenograft control mice**								
**Number of peaks (*m*/*z*)**	**Prediction in test set**		**Test performance**					
	**No. of correct results**	**No. of incorrect results**	**Sensitivity (%)**	**Specificity (%)**	**PPV (%)**	**NPV (%)**		**Efficiency (%)**
22	19	1	92.3	100	100	87.5		95.0
								
**(b) Treated *vs* untreated OE19 xenograft-bearing mice**								
**Drug**		**Prediction in test set**		**Test performance**				
	**No. of peaks (*m*/*z*)**	**No. of correct results**	**No. of incorrect results**	**Sensitivity (%)**	**Specificity (%)**	**PPV (%)**	**NPV (%)**	**Efficiency (%)**
**Epirubicin**	9	11	0	100	100	100	100	100
**Cisplatin**	7	10	1	100	85.7	80.0	100	90.9
**5-FU**	14	8	1	100	85.7	66.7	100	88.9

Abbreviations: 5-FU=5-fluorouracil; PPV=positive predictive value; NPV=negative predictive value.

**Table 2:** Identification of significant proteins

***m*/*z***	**Predictive for^a^**	**Protein (mouse)**	**MOWSE score**	**No. of peptides**	**Sequence covered (%)**
8880	4	Apolipoprotein C-III	1048	24	64
11 680	2	*β-*2-microglobulin	384	22	57
11 750	1	Serum amyloid A	358	14	63
14 040	1, 2, 3	Transthyretin	271	5	24
15 830	1	Haemoglobin *β*-2	648	14	82
16 020	1	Haemoglobin *β*-1	1145	20	84
28 880	1, 2, 4	Apolipoprotein A-I	731	20	50

a 1=xenograft *vs* non-xenograft; 2=epirubicin-treated *vs* -untreated xenograft; 3=cisplatin-treated *vs* untreated xenograft; 4=5-fluorouracil-treated vs -untreated xenograft.

For each peak (*m*/*z*), the treatment for which the peak was predictive, the protein identified, the MOWSE probability score and sequence coverage is shown.

**Table 3:** Identification of significant proteins in clinical samples

***m*/*z***	**Protein (mouse)**	**Approx. MW**	**MOWSE score**	**No. of peptides**	**Sequence covered (%)**
11 600	Serum amyloid A	11 700	1131	37	69
14 000	Transthyretin	14 000	2181	66	83
21 000	Retinol-binding protein 4	21 100	1050	46	77
28 200	Apolipoprotein A-I	28 000	1854	58	42

Abbreviation: MW=molecular weight. For each peak (*m*/*z*), the protein identified is shown, together with the expected MW in plasma, the MOWSE probability score and sequence coverage.
